# Effect of Antazoline on Electrophysiological Properties of Atrial Muscle and Conduction System of the Heart

**DOI:** 10.1007/s10557-018-6787-9

**Published:** 2018-04-06

**Authors:** Bartłomiej Jacek Bińkowski, Marcin Makowski, Paweł Kubiński, Andrzej Lubiński

**Affiliations:** Department of Invasive Cardiology and Cardiac Arrhythmias, University Clinical Hospital Military Memorial Medical Academy - Central Veterans’ Hospital in Lodz, Żeromskiego 113, 90-549 Łódź, Poland

**Keywords:** Antazoline, Antiarrhythmic drug, Electrophysiological study

## Abstract

**Purpose:**

Antazoline is a first-generation antihistaminic agent with additional anticholinergic properties and antiarrhythmic potential. Recent data shows its high effectiveness in sinus rhythm restoration among patients with paroxysmal atrial fibrillation. The effect of antazoline on electrophysiological parameters of the heart in vivo has not yet been examined. The aim of this study was to evaluate changes in electrophysiological parameters of the heart muscle and conduction system as a response to increasing doses of antazoline.

**Methods:**

After successful ablation of supraventricular arrhythmias, the electrophysiological parameters: sinus rhythm cycle length (SRCL), AH, HV, QRS, QT, QTc intervals, Wenckebach point (WP), sinus node recovery period (SNRT), intra- (hRA-CSos) and interatrial conduction time (hRA-CSd), right and left atrium refractory period (RA-; LA-ERP), and atrioventricular node refractory period (AVN-ERP) were assessed initially and after 100, 200, and 300 mg of antazoline given intravenously.

**Results:**

Fifteen patients (8 males, 19–72 years old) undergoing EPS and RF ablation were enrolled. After 100 mg bolus, a significant reduction in SRCL was noticed. After antazoline administration, significant prolongation of HV, QRS, QTc, hRA-CSos, hRA-CSd intervals, RA– and LA-ERP and reduction of SRCL were observed. After a total dose of 300 mg, QT interval prolonged significantly. Increasing the dose of antazoline had no impact on AH, Wenckebach point, AVN-ERP, and SNRT.

**Conclusion:**

Antazoline has an effect on electrophysiological parameters of the atrial muscle and has rapid onset of action. No negative effect on sinus node function and atrioventricular conduction in a unique property among antiarrhythmic drugs.

## Introduction

Antazoline is a first-generation antihistaminic agent with additional anticholinergic properties, first described in 1947 [1]. Its antiarrhythmic potential was first discovered in the 1950s of the last century [2–4]. In subsequent years, the effects of antazoline administered both orally and intravenously on different types of arrhythmias were assessed. There were antiarrhythmic effects against premature atrial and ventricular beats, nodal reentrant tachycardia and atrial and ventricular tachycardia. Initially, poor effect was noticed against atrial fibrillation (AF), probably because the great part of sinus rhythm restoration attempts was made among patients with longstanding persistent arrhythmia [5–8]. Only one paper described 66% success rate in AF treatment [9]. Moreover, complications from oral use of antazoline, mainly immunological and hematological, caused its widespread chronic use as an antiarrhythmic agent to be abandoned [10]. In 2000, new data on the efficacy of antazoline was published. Retrospective analysis of 1325 patients treated with intravenous antazoline for paroxysmal AF revealed that antazoline restored sinus rhythm in 52% of the patients [11]. Recent data indicate an even higher success rate approaching 80%. The superiority of the antazoline over placebo in sinus rhythm restoration in recent onset AF has been confirmed in the AnPAF study. Complications of ad hoc administered antazoline seem to be rare and benign [12–15]. The effect of antazoline on electrophysiological parameters of the heart in vivo has not yet been examined. Thus, the aim of this study was to evaluate changes in electrophysiological parameters of the heart muscle and conduction system as a response to increasing doses of antazoline.

## Methods

### Patient Population

After obtaining approval from the Ethics Committee (RNN/584/11/KB; 12.07.2011), we enrolled patients undergoing electrophysiological study for paroxysmal arrhythmias: atrioventricular nodal reentrant tachycardia (AVNRT), atrioventricular reentrant tachycardia (AVRT), and common atrial flutter (AFL). The main inclusion criteria were successful radiofrequency (RF) ablation of underlying arrhythmia and stable therapeutic effect of the ablation lasting for at least 15 min since last RF application. We included patients over the age of 18 without concomitant disease. Patients were off the antiarrhythmic drugs for at least five drug half-lives. Exclusion criteria were abnormal echocardiography findings (chambers dimensions, systolic and diastolic function of the ventricles, greater than mild valve disorders), use of amiodarone within last 3 months, use of drugs affecting QT interval, abnormal lab tests (full blood count, GFR, plasma electrolytes, ALT), need for use of drugs others than unfractionated heparin during the ablation (isoproterenol, atropine, ephedrine, antiarrhythmics, etc.), or recurrence of underlying arrhythmia. Written informed consent was obtained from each patient before entry into the study.

### Electrophysiological Study

For electrophysiological study (EPS), three catheters were introduced through the right femoral vein and placed in high right atrium (hRA), coronary sinus (CS), and His bundle region (HIS). EPS evaluated the sinus rhythm cycle length (SRCL) averaged from 10 consecutive cycles, duration of AH, HV, QRS, QT, QTc (Bazett formula) intervals taken on sinus rhythm, Wenckebach point (WP), sinus node recovery period (SNRT), intra- and inter atrial conduction times measured as hRA- CS ostium interval (hRA-CSos) and hRA-CS distal interval (hRA-CSd), right and left atrium refractory period (RA-ERP, LA-ERP), and atrioventricular node refractory period (AVN-ERP). After initial measurements, antazoline was administered in 100 mg/3 min-boluses up to a total dose of 300 mg. The dose and administration rate were based on doses recommended for arrhythmia treatment in Summary of Product Characteristics. Five minutes after initiation of every antazoline bolus, all of the measurements were repeated, except SNRT which was taken initially and after the full dose of antazoline. The time interval between the beginning of each injection was 10 min. During the entire EPS, blood pressure and saturation were monitored noninvasively. After the study, ECG was monitored for at least 2 h.

### Statistical Analysis

The Shapiro-Wilk test was used to assess distribution of data. Data is shown as mean and standard error of the mean (SEM). Statistical analysis was performed using non-parametric statistics. Wilcoxon matched pair signed-rank test was used to compare two time points. Friedman’s and Dunn’s test was used to compare four points in the same group. Statistical significance was defined as *p* < 0.05.

## Results

**Fig. 1:**
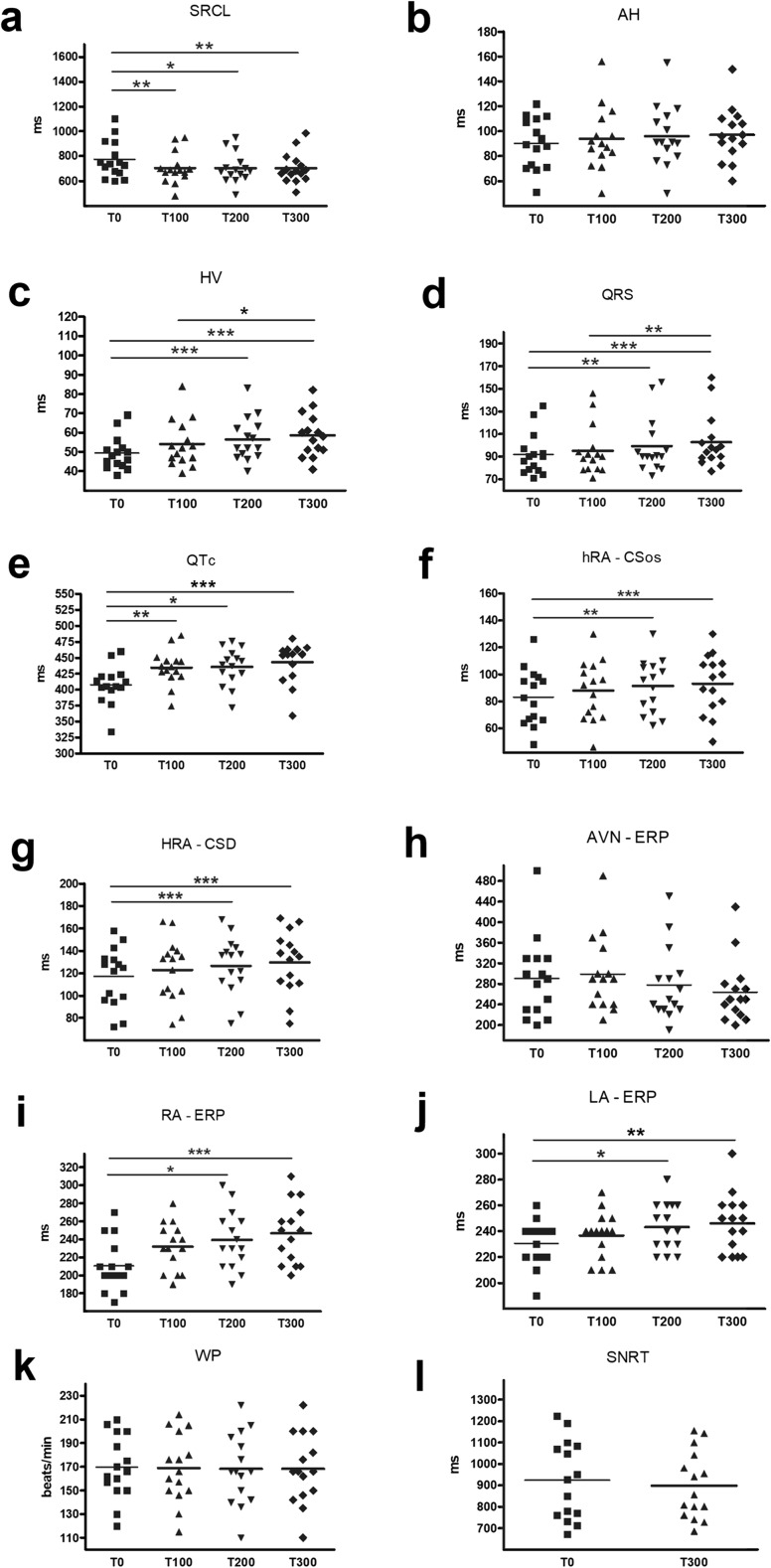
Effect of antazoline on electrophysiological properties of atrial muscle and conduction system of the heart. SRCL, sinus rhythm cycle length; hRA-CSos, intraatrial conduction time; hRA-CSd interatrial conducion time; RA-ERP right atrium refractory period; LA-ERP, left atrium refractory; AVN-ERP, atrioventricular node refractory period; WP, Wenckebach point; SNRT, sinus node recovery period; T0 initial value, T100 after 100, T200 after 200, T300 after 300 mg of antazoline. *p < 0.05, **p < 0.01, ***p < 0.001

We enrolled 15 patients (8 males, 19–72 years old, EF 61 ± 2.5%) undergoing EPS and RF ablation. The indications for EPS were AVNRT (*n* = 6), AVRT (*n* = 4), and AFL (*n* = 5). After 100 mg bolus, a significant reduction in SRCL and QTc prolongation was noticed. After a total dose of 200 mg, we observed significant prolongation of HV, QRS, hRA-CSos and hRA-CSd intervals and RA- and LA-ERP. After a total dose of 300 mg, QT interval prolonged significantly. Increasing the dose of antazoline had no impact on AH, WP, AVN-ERP, and SNRT. The results are summarized in Table 1 and on Fig. 1. In one patient, after total dose of 300 mg of antazoline, we induced common atrial flutter with incremental atrial pacing. Arrhythmia cycle length was 200 ms with 2:1 atrioventricular conduction. The basic EPS finding in this patient was AVNRT and there was no evidence of pre-existing common atrial flutter. Arrhythmia was terminated with overdrive atrial pacing. After full dose of antazoline, two patients reported hot flush sensation and one nausea that resolved without any intervention.

**Table 1:** Results summary. *SRCL* sinus rhythm cycle length, *hRA-CSos* intraatrial conduction time, *hRA-CSd* interatrial conducion time, *RA-ERP* right atrium refractory period, *LA-ERP* left atrium refractory, *AVN-ERP* atrioventricular node refractory period, *WP* Wenckebach point, *SNRT* sinus node recovery period, *BPsys* systolic blood pressure, *BPdias* diastolic blood pressure

	Baseline	100 mg	*P*	200 mg	*P*	300 mg	*P*
SRCL (ms)	772.8 ± 38.47	703.4 ± 32.51	< 0.01	703.5 ± 31.03	< 0.05	702.9 ± 31.31	< 0.01
AH (ms)	90.26 ± 5.28	93.93 ± 6.47	ns	96.06 ± 6.34	ns	97.13 ± 5.59	ns
HV (ms)	49.3 ± 2.22	54 ± 3.09	ns	56.46 ± 2.89	< 0.001	58.53 ± 2.89	< 0.001
QRS (ms)	91.93 ± 4.84	95.13 ± 5.64	ns	99.27 ± 6.43	< 0.01	102.73 ± 6.21	< 0.001
QT (ms)	356 ± 7.54	363.33 ± 7.87	ns	363.33 ± 7.29	ns	369.13 ± 7.20	< 0.001
QTc (ms)	408.07 ± 38.18	434.67 ± 9.89	< 0.05	435.73 ± 20.51	< 0.01	443 ± 3.53	< 0.001
hRA-Csos (ms)	83.2 ± 5.39	87.87 ± 5.68	ns	91.4 ± 5.1	< 0.01	93.13 ± 5.67	< 0.001
hRA-CSd (ms)	117.13 ± 6.75	122.8 ± 7.13	ns	126.67 ± 6.64	< 0.001	129.73 ± 7.15	< 0.001
AVN-ERP (ms)	290.67 ± 20.15	298.67 ± 18.97	ns	278 ± 18.24	ns	252 ± 22.39	ns
RA-ERP (ms)	210.67 ± 7.27	232 ± 6.77	ns	239.33 ± 8.31	< 0.05	246.67 ± 8.71	< 0.001
LA-ERP (ms)	230.67 ± 4.52	236.67 ± 4.65	ns	243.33 ± 4.75	< 0.05	246 ± 5.84	< 0.01
WP (bpm)	169.53 ± 6.99	168.73 ± 7.49	ns	168.2 ± 7.71	ns	168.2 ± 7.63	ns
SNRT (ms)	924.2 ± 47.55	N/A		N/A		899.53 ± 40.60	ns
BPsys (mmHg)	123.46 ± 15.55	N/A		N/A		120.47 ± 13.43	ns
BPdias (mmHg)	71.33 ± 17.67	N/A		N/A		73 ± 15.55	ns

*N/A* not available, *ns* not significant

## Discussion

To our knowledge, this is the first study assessing effect of antazoline on electrophysiological parameters of the human heart. The data on antazoline effectiveness in ad hoc AF termination is constantly growing. After several nonrandomized observational studies the result of randomized clinical trial comparing antazoline and placebo for sinus rhythm restoration in paroxysmal AF has been published showing high effectiveness in sinus rhythm restoration exceeding 70%. The rate of spontaneous sinus rhythm recoveries in placebo arm was only 10% [15]. While chronic antiarrhythmic therapy is practically impossible due to serious complications, it is still a very attractive option for rapid AF termination and in some countries it is registered and widespread used for this purpose.

Our results clearly indicate influence of antazoline on heart conduction system and muscle. Measured parameter changes: HV prolongation, QRS widening, atrial refractory period prolongation, and conduction velocity slowing are similar to agents classified as class I of Vaughan Williams classification of antiarrhythmic drugs. The constellation of changes of parameters in EPS suggests an influence of antazoline mainly on sodium channels. Moreover, known anticholinergic properties place antazoline near class Ia agent: quinidine. This data is consistent with the ELEPHANT study results. The early effect on surface ECG is similar to sodium channel blocker. In the ELEPHANT study, observation time was longer and revealed potential potassium channels inhibition and more pronounced QT interval prolongation in the later phase of observation [16]. In our group observation, period was probably too short to disclose these changes, but QT prolongation was also noticed.

QTc prolongation is very important finding of our study. QTc change resulted mainly from rhythm acceleration without simultaneous absolute QT interval shortening. Many patients with paroxysmal AF are treated for rhythm control with medications prolonging QT interval, mainly class III antiarrhythmic drugs. It is necessary to keep caution in these patients or consider abandoning use of antazoline for sinus rhythm restoration.

Our observations confirm another important property of antazoline: rapid onset of action. We noticed significant influence on electrophysiological parameters in measurements obtained after a total dose of 200 mg and 20 min from the beginning of first bolus. Shortening of SRCL appeared almost immediately, after first bolus, and remained at the same level after further doses. This effect apparently results from rapid anticholinergic action of antazoline. Clinical data showed short time to AF cessation during WPW ablation. From the beginning of antazoline infusion, it took on average 7 min to restore sinus rhythm ^12^. In the AnPAF study, mean time to sinus rhythm restoration was only 16 min [15]. This period could have been shorter than that required to significantly change the electrophysiological properties of the atria in our observation and only the SRCL has changed this early; however, the antazoline administration protocol differed between the studies. Thus, inhibition of parasympathetic ganglia may play an important role in quick AF cessation. Among antiarrhythmic drugs, only the recently approved vernakalant is comparably fast in sinus rhythm restoration [17, 18]. Other commonly used agents usually require hours to achieve AF termination.

One of the most common side effects of antiarrhythmic therapy is bradycardia related to sinus node dysfunction or conduction disturbances. We did not notice any negative effects of antazoline either on sinus node function nor atrioventricular conduction. It has even accelerated sinus rhythm. Thanks to such properties, it may potentially become the drug of choice in patients with tachycardia—bradycardia syndrome, diagnosed sinus node dysfunction, or impaired atrioventricular conduction not protected with permanent pacemaker.

Our observations, together with previous studies on antazoline efficacy, build a basis for large scale, randomized trials on antazoline as a drug of choice for rapid, ad hoc treatment of recent onset AF.

### Study Limitations

Patients with paroxysmal arrhythmias and after arrhythmia induction in EP study may response differently to antazoline in comparison to healthy people. Study involving healthy volunteers would be impossible to conduct due to ethical issues. Plasma drug levels were not assessed in the study, thus, association between electrophysiological parameters and drug concentration is unknown.

## Conclusion

Antazoline has an effect on electrophysiological parameters of the atrial muscle and has rapid onset of action. No negative effect on sinus node function and atrioventricular conduction in a unique property among antiarrhythmic drugs.
